# Identification of a methylation panel as an alternative triage to detect CIN3+ in hrHPV-positive self-samples from the population-based cervical cancer screening programme

**DOI:** 10.1186/s13148-023-01517-6

**Published:** 2023-06-15

**Authors:** J. de Waard, A. Bhattacharya, M. T. de Boer, B. M. van Hemel, M. D. Esajas, K. M. Vermeulen, G. H. de Bock, E. Schuuring, G. B. A. Wisman

**Affiliations:** 1 Department of Gynaecologic Oncology, Cancer Research Center Groningen, University of Groningen, University Medical Center Groningen, PO-Box 30001, 9700 RB Groningen, The Netherlands; 2 Department of Medical Oncology, Cancer Research Center Groningen, University of Groningen, University Medical Center Groningen, Groningen, The Netherlands; 3 Department of Pathology, University of Groningen, University Medical Center Groningen, Groningen, The Netherlands; 4 Department of Obstetrics and Gynaecology, University of Groningen, University Medical Center Groningen, Groningen, The Netherlands; 5 Department of Epidemiology, University of Groningen, University Medical Center Groningen, Groningen, The Netherlands

**Keywords:** Self-sampling, Cervical cancer screening, DNA methylation markers, Quantitative methylation-specific PCR (QMSP), hrHPV, Cervical intraepithelial neoplasia (CIN)

## Abstract

**Background:**

The Dutch population-based cervical cancer screening programme (PBS) consists of primary high-risk human papilloma virus (hrHPV) testing with cytology as triage test. In addition to cervical scraping by a general practitioner (GP), women are offered self-sampling to increase participation. Because cytological examination on self-sampled material is not feasible, collection of cervical samples from hrHPV-positive women by a GP is required. This study aims to design a methylation marker panel to detect CIN3 or worse (CIN3+) in hrHPV-positive self-samples from the Dutch PBS as an alternative triage test for cytology.

**Methods:**

Fifteen individual host DNA methylation markers with high sensitivity and specificity for CIN3+ were selected from literature and analysed using quantitative methylation-specific PCR (QMSP) on DNA from hrHPV-positive self-samples from 208 women with CIN2 or less (< CIN2) and 96 women with CIN3+. Diagnostic performance was determined by area under the curve (AUC) of receiver operating characteristic (ROC) analysis. Self-samples were divided into a train and test set. Hierarchical clustering analysis to identify input methylation markers, followed by model-based recursive partitioning and robustness analysis to construct a predictive model, was applied to design the best marker panel.

**Results:**

QMSP analysis of the 15 individual methylation markers showed discriminative DNA methylation levels between < CIN2 and CIN3+ for all markers (*p* < 0.05). The diagnostic performance analysis for CIN3+ showed an AUC of ≥ 0.7 (*p* < 0.001) for nine markers. Hierarchical clustering analysis resulted in seven clusters with methylation markers with similar methylation patterns (Spearman correlation> 0.5). Decision tree modeling revealed the best and most robust panel to contain ANKRD18CP, LHX8 and EPB41L3 with an AUC of 0.83 in the training set and 0.84 in the test set. Sensitivity to detect CIN3+ was 82% in the training set and 84% in the test set, with a specificity of 74% and 71%, respectively. Furthermore, all cancer cases (*n* = 5) were identified.

**Conclusion:**

The combination of ANKRD18CP, LHX8 and EPB41L3 revealed good diagnostic performance in real-life self-sampled material. This panel shows clinical applicability to replace cytology in women using self-sampling in the Dutch PBS programme and avoids the extra GP visit after a hrHPV-positive self-sampling test.

**Supplementary Information:**

The online version contains supplementary material available at 10.1186/s13148-023-01517-6.

## Introduction

The introduction of population-based screening (PBS) programmes has led to a significant reduction in the incidence and mortality of cervical cancer [1–3]. In the Netherlands, since 1996 women aged 30–60 years get an invitation every 5 years to participate in the PBS [4, 5]. This Dutch PBS used to be cytology based, but in 2017 changed to primary high-risk human papilloma virus (hrHPV) testing, because hrHPV testing has a significantly higher sensitivity than cytology [5–8]. However, the specificity of the hrHPV test is modest, as it cannot distinguish between transient and progressive hrHPV infections [9, 10]. To avoid unnecessary referrals to the gynaecologist, cytology is performed as triage test in hrHPV-positive cases. One drawback of cytology is its subjective interpretation [11–13].

Since 2017, instead of participating by visiting the general practitioner (GP) for a cervical smear, women in the Netherlands also have the option to receive and use a self-sampling device [14]. The use of a self-sampling device is more convenient than visiting a GP, as it can be performed at home in one’s own time [15]. However, it also comes with some disadvantages, as cytology cannot be performed on self-samples [16, 17]. Consequently, hrHPV-positive women still need to visit a GP for a cytological examination to evaluate whether a referral to a gynaecologist is indicated. This extra GP visit is often experienced as unwelcome and results in lower compliance [18]. In 2021, 22.1% of the women participating in the PBS used a self-sampling device, of these women 8.4% were hrHPV-positive (~ 10,000 women) [19]. However, between 2017 and 2020 only 90% of the women with an hrHPV-positive test result visited the GP for cytological examination within 27 months [17]. The remaining 10% of the women that received an hrHPV-positive test result did not visit their GP, even though they were at risk for CIN3+. A triage test performed on hrHPV-positive self-samples could diminish an extra GP visit and reduce the loss to follow-up at the same time.

Increased promoter DNA methylation of several tumour suppressor genes plays an important role in the development of cervical cancer [20–22]. Numerous studies have shown that detection of DNA methylation of one or more host genes can be used as an objective triage screening method for hrHPV-positive women [23–28]. However, the majority of studies is performed on cervical smears taken by a GP obtained in cohorts that do not reflect the population participating in the PBS. In addition, most of the methylation markers are analysed in separate studies and only few compared the performance of some markers simultaneously.

Our aim was to identify a panel of methylation markers with the highest sensitivity and specificity to detect CIN3+ in material collected with the self-sampling device. This panel can be directly applied on self-samples and will finally result in avoiding an extra physician or GP visit for many women worldwide. For this purpose, we selected 15 promising host DNA methylation markers based on a systematic literature search (see Additional file 1: Table S1) and tested in this study for the first time all discriminative methylation markers to detect CIN3+ within the same cohort, consisting of hrHPV-positive self-samples collected within the Dutch PBS.

## Materials and methods

### Selection of methylation markers

A systematic Pubmed/Medline, Embase and Cochrane literature search was performed to identify relevant studies that analysed methylation markers in cervical specimens until November 1, 2019 (manuscript in prep.). Hereafter, 15 methylation markers that fulfilled the following criteria (Additional file 1: Table S1) were included in this study: 1. sensitivity and specificity to detect CIN3+ of ≥ 70% and ≥ 60% (individually or as a panel) in at least one study, 2. markers tested on a cohort of > 50 cervical samples, 3. histology used as the gold standard test for diagnosis, 4. the use of QMSP, 5. primer/probe sequences available (commercially available markers were excluded).

### Study population

Local biobank permission was obtained according to the local and National Institute for Health and the Environment (RIVM) regulations to collect samples obtained within the PBS. Samples from women (aged 30–60) were selected for this study who participated in the PBS in the North of the Netherlands in the period from December 2018 until May 2020 using a self-sampling device (Evalyn Brush, Rovers Medical Devices B.V., Oss, the Netherlands) and had an hrHPV-positive result using Cobas® 4800 HPV test (Roche Diagnostics, Alameda CA, USA). Histology of the biopsy taken by the gynaecologist was used as the gold standard. Histology results were retrieved at the nationwide network and registry of histo- and cytopathology in the Netherlands (PALGA Foundation). Histology was categorized as CIN0, CIN1, CIN2, CIN3 and cancer. Women with two consecutive normal cytology results (at primary screening [*t* = 0 months] and 6 months of follow-up screening [control smear], and therefore not referred to the gynaecologist for colposcopy) were considered as hrHPV-positive women without disease (i.e. negative for intraepithelial lesion or malignancy [NILM]). All consecutive CIN2+ cases were selected, of the same period women with proven NILM, CIN0/1 were randomly selected.

### DNA isolation

The self-samples were first used for hrHPV testing (using the Cobas®4800 HPV test) in the national PBS and the residual material in ~ 20 ml ThinPrep preservation medium (Hologic Inc., Marlborough, MA, USA) was stored at room temperature. For this study, four milliliter of ThinPrep was used, and cells were pelleted by centrifugation for 10 min at 500 g. DNA was isolated by standard overnight 1% SDS and proteinase K treatment, salt-chloroform extraction and isopropanol precipitation. DNA pellets were washed with 70% ethanol and dissolved in 100 µl TE^−4^ buffer (10 mM Tris/HCL; 0.1 mM EDTA, pH 8.0) [29]. DNA concentration was measured using Qubit fluorometer with the dsDNA BR assay kit (ThermoFisher Scientific, Waltham, MA, USA) according to the manufacturer's instructions. DNA quality control was performed by the BIOMED-2 protocol and visualized by gel electrophoresis [30].

### Quantitative methylation-specific PCR

Prior to QMSP, sodium bisulfite treatment on isolated genomic DNA (600 ng per sample) was performed according to the manufacturer's protocol of the EZ DNA methylation kit (Zymo Research Corp, Irvine, CA, USA), except that elution was done with 100 µl M-Elution Buffer yielding approximately 6 ng/µl bisulfite-converted DNA.

QMSP was performed with bisulfite-treated DNA using an internal (FAM-ZEN/IBFQ)-labelled hybridization probe (IDT, Leuven, Belgium) for quantitative analyses of the 15 different methylation markers (for list see Additional file 1: Table S1)*.* Primer and probe sequences are available upon request. The housekeeping gene β-actin was used to correct for DNA input [31]. QMSP reactions were performed in a total volume of 30 µl, containing QuantiTect Probe Mastermix (Qiagen, Hilden, Germany), 10 µM of forward and reverse primers (Invitrogen, Carlsbad, CA, USA), 5 µM hybridization probe (IDT, Leuven, Belgium) and 45 ng bisulfite-treated DNA. Each sample was analysed in a 96-well plate using the Abbott m2000rt System (Abbott Molecular Inc., Des Plaines, IL, USA) with the following conditions: 10 min at 95 °C followed by 50 cycles of 10 s at 95 °C and 1 min at 60 °C. As a methylation positive control, serial dilutions of in vitro methylated genomic DNA with Sss I (CpG) methyltransferase (New England Biolabs, Beverly, MA, USA) were used in each run. H_2_O and whole genome amplified (WGA) of leucocyte DNA using the illustra™ Ready-To-Go™ GenomePhi™ high yield (HY) DNA amplification kit (GE healthcare, Chicago, IL, USA) were used as methylation negative controls. All amplification curves were reviewed and scored without knowledge of clinical data. A sample was considered invalid if the Ct-value for β-actin was ≥ 32. QMSP values (∆Ct values) were adjusted for DNA input by expressing results as ratios between two absolute measurements (Ct value marker – Ct value β-actin). Methylation levels were calculated using the formula 2^−∆Ct * 100. Negative samples (no Ct value before 50 cycles) were assigned a ∆Ct of 30.

### Statistical analyses

Statistical analysis was performed using SPSS software package (SPSS 28, Chicago, IL, USA) and RStudio software (version 1.4.1106). ROC curves were generated based on ∆Ct values to detect CIN2+ and CIN3+ as cut-off, and the AUC was used as a measure of model performance. Kruskal–Wallis test and Mann–Whitney *U* test were performed to identify differences in methylation levels among two groups or more. Differences in results were considered statistically significant when the *p*-value was < 0.05. Graphical representations were created with GraphPad Prism 9 or RStudio software (version 1.4.1106).

### Hierarchical clustering analysis

To evaluate which methylation markers showed similar methylation patterns, hierarchical clustering based on Spearman correlation was performed with all 15 markers.

A data frame was created from the QMSP data with the ∆Ct values of methylation markers for all samples. 1-Spearman correlation between ∆Ct values of methylation markers was used as distance matrix with ward.D2 method for the hierarchical clustering analysis. The hclust function from stats package version 4.0.5 in RStudio was used [32, 33], to perform hierarchical clustering analysis on ∆Ct values of methylation markers, and a dendrogram was obtained. The height cut-off level in the dendrogram to define markers being member of the same cluster was set at 0.5. Heatmaps were created using the heatmap.2 function from gplots package version 3.1.1.

### Model-based recursive partitioning to construct a predictive model

To identify a panel with the most discriminative methylation markers for the detection of CIN3+, model-based recursive partitioning was applied. For a detailed description of the model-based recursive partitioning see Additional file 1: Methods. In short, model-based recursive partitioning (MOB) with the mob function from the party package version 1.3–9 in R was used [34], to create a decision tree model with the most discriminative panel of methylation markers to detect CIN3+. The samples and corresponding ∆Ct values for all the tested markers were divided using stratified random sampling based on histological diagnosis (without replacement) into a train (80%) and test set (20%) to ensure that both sets contained an equal percentage of CIN3+ samples. MOB was separately conducted using all possible combinations of markers (based on the clustering analysis) with a minimum of one to a maximum of six as predictors. The models that fulfilled the following criteria were selected for further investigation: sensitivity > 80% and specificity > 65% and MCC > 0.5 in the test set. Subsequently, a robustness analysis was performed separately for those models which fulfilled selection criteria. MOB was conducted 1000 times using random 80% of the samples as training set and 20% as test set each time.

Robust models were selected, using the following criteria: a robustness score of above 500 out of 1000 for the classifier, all the individual predictors needed to have a robustness score of above 500 out of 1000, the mean of the standard deviations of the coefficients needed to be below 1.

## Results

### Study cohort

**Fig. 1:**
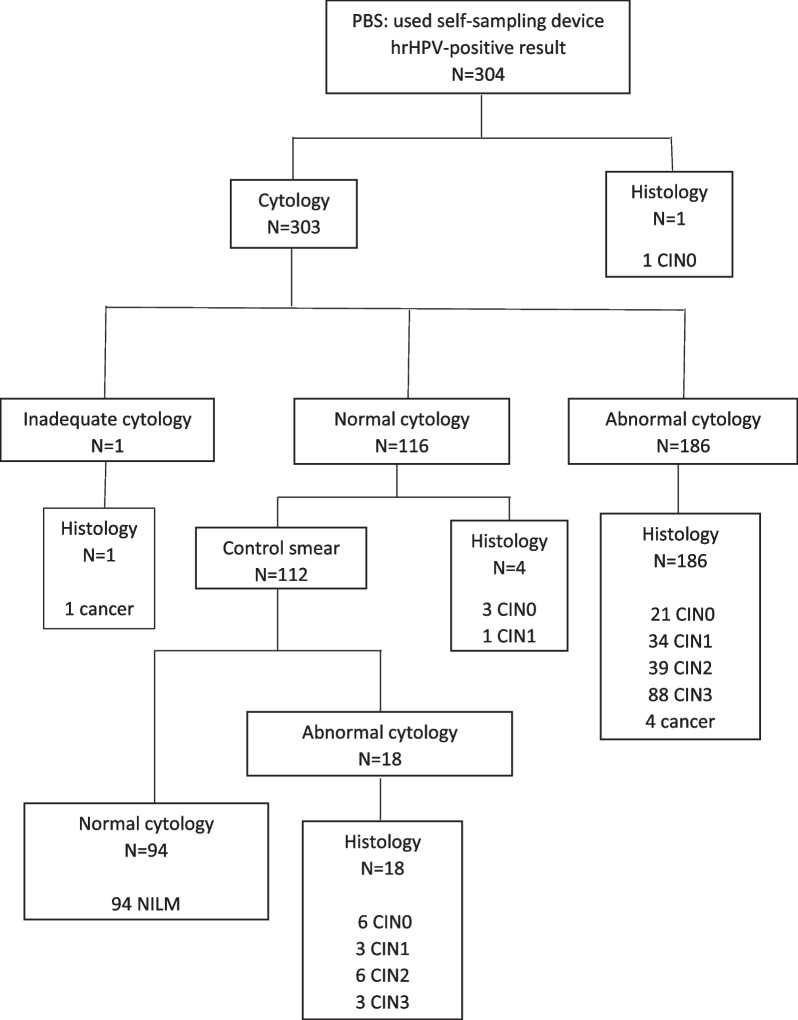
Study population. Samples eligible for the study based on quality control criteria

The following samples were included in this study: NILM (2 × normal cytology, *N* = 110); CIN0 (*N* = 35); CIN1 (*N* = 39); CIN2 (*N* = 50); CIN3 (*N* = 96); cancer (*N* = 5). The mean age of the women per group is: NILM 40.5 years (95% CI 38.5–42.4), CIN0: 40.3 years (95% CI 36.7–43.9), CIN1: 35.0 years (95% CI 33.0–37.0), CIN2: 35.6 years (95% CI 33.4–37.7), CIN3: 35.4 years (95% CI 34.0–36.8), cancer: 36.4 years (95% CI 30.5–42.3). DNA quality control showed that 91% of all samples yielded sufficient amount of high-quality DNA to perform methylation analysis (7.5% of the samples did not contain sufficient DNA required to perform bisulfite treatment (> 600 ng), 1.8% β-actin Ct above 32). This resulted in 304 included samples in this study (Fig. 1). One woman directly underwent histology after receiving an hrHPV-positive result and was diagnosed with CIN0. At baseline cytology, 186 women had an abnormal result, of whom 92 had CIN3+ as outcome. One hundred and sixteen women had a normal cytology result, of whom 94 had a control smear with a normal cytology result. Eighteen had a control smear with an abnormal cytology result, of whom three had CIN3 as outcome and six CIN2. One woman received an inadequate cytology result and directly underwent histology and was diagnosed with cervical cancer. The five cancer cases included in this study were classified as two adenocarcinomas and three squamous cell carcinomas.

### Performance of the individual methylation markers

**Fig. 2:**
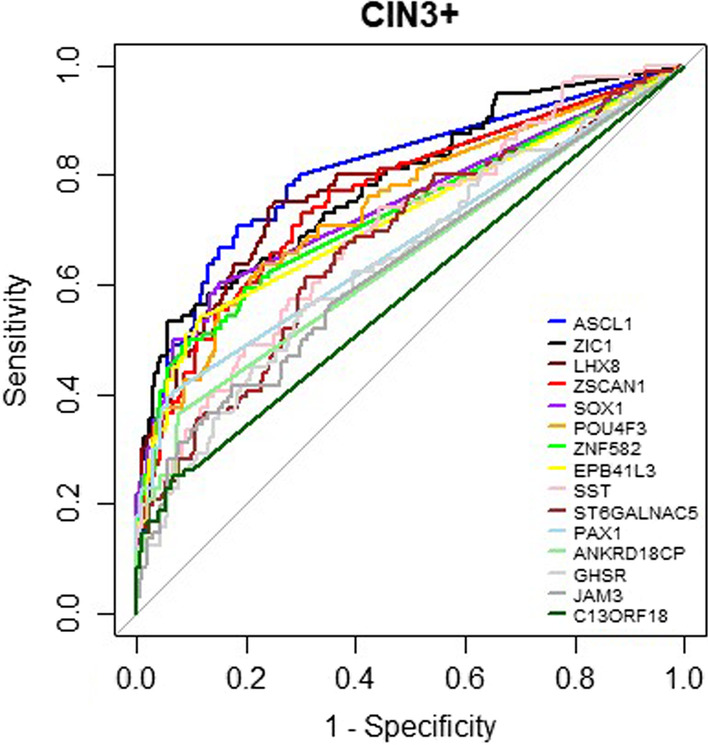
ROC curves for ∆Ct values of the 15 individual methylation markers for the detection of CIN3+

To evaluate the diagnostic performance of the 15 individual methylation markers, methylation levels of each marker were analysed separately across the different histological subgroups (NILM *N* = 94, CIN0 *N* = 31, CIN1 *N* = 38, CIN2 *N* = 45, CIN3 *N* = 91, cancer *N* = 5). Methylation levels of all 15 markers increased with the severity of underlying lesions (*p* < 0.05) (Additional file 1: Fig. S1). Each of the 15 markers was highly significantly discriminative between the CIN3+ and < CIN3 lesions (*p* < 0.001). ROC analysis to measure diagnostic performance of the separate markers for CIN3+ showed that 9/15 markers had an AUC of ≥ 0.7 (*p* < 0.001) (Fig. 2, Additional file 1: Table S2). In addition, the same 15 markers were also discriminative between CIN2+ and < CIN2 (*p* < 0.05) and ROC analysis for CIN2+ showed 4/15 markers had an AUC of ≥ 0.7 (Additional file 1: Fig. S2, Additional file 1: Table S2).

### Creating the most optimal methylation marker panel

**Fig. 3:**
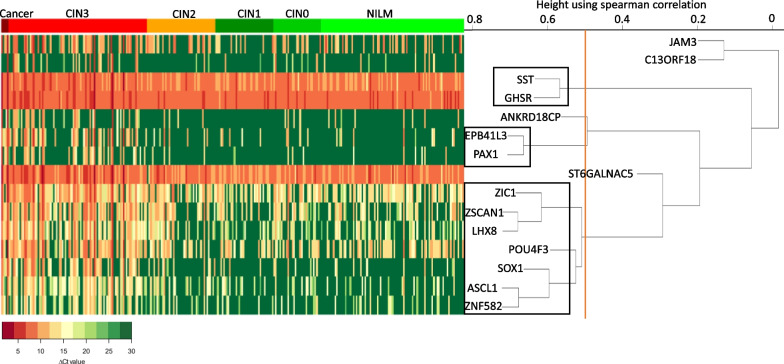
Hierarchical clustering of the methylation markers. The rows represent the different methylation markers, and the columns the individual hrHPV-positive self-samples. The self-samples are ranked based on histological outcome (legend on top). The colours in the histogram demonstrate the ∆Ct values, green represents a high ∆Ct (low methylation level) and red a low ∆Ct (high methylation level). The cut-off of the Spearman correlation to identify markers present in the same cluster was set at 0.5 (indicated by the orange line). The black boxes represent the different clusters

To further increase the discriminative ability between CIN3+ and < CIN3, we next explored the effect when combining different methylation markers. With these selected 15 methylation markers, numerous combinations are possible. Assuming that markers with a similar methylation pattern do not have additional diagnostic value, prior to evaluating various combinations, we first performed hierarchical clustering based on Spearman correlation with all 15 markers in our cohort of 304 hrHPV-positive self-samples (Fig. 3). This cluster analysis revealed several methylation markers with a very similar methylation pattern, as for example EPB41L3 and PAX1, as well as ZSCAN1 and LHX8. After setting a cut-off for the Spearman correlation at 0.5 to identify markers present in the same cluster, the analysis resulted in three different clusters with more than one marker and four clusters with one marker. Two of the clusters consisted of two markers (*SST* and *GHSR*, and *EPB41L3* and *PAX1)*. One cluster consisted of seven markers (*ZIC1, ZSCAN1, LHX8, POU4F3, SOX1, ASCL*1 and *ZNF582*). Four clusters consisted of only one marker (*JAM3, C13ORF18, ANKRD18CP* and *ST6GALNAC5*).

**Fig. 4:**
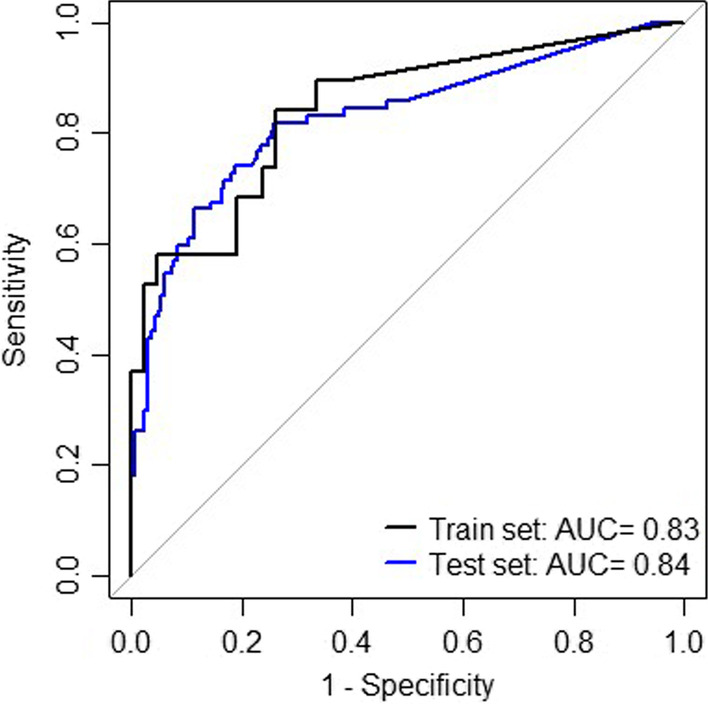
ROC curves of the decision tree model to detect CIN3+. The decision tree model consists of the markers ANKRD18CP, LHX8 and EPB41L3. The AUC is calculated on the train and the test set

**Fig. 5:**
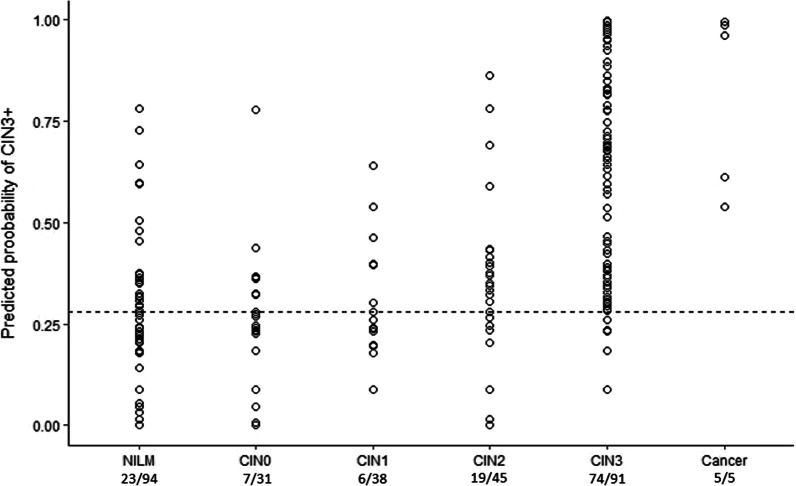
Predicted probability of CIN3+ per histological subgroup. The decision tree model was used to obtain the predicted probability for the different self-samples based on the full data. The cut-off of the predicted probability to consider a sample CIN3+ based on the ROC curve of the train set and Youden index is 0.28

Second, a decision tree model was created using model based recursive partitioning on the ∆Ct values of the methylation markers obtained from the QMSP results. There were 1,123 possible combinations of minimal one and maximal six markers, which were not members of the same cluster. From these 1,123 combinations, seven models fulfilled our set criteria with sensitivity > 80% and specificity > 65% and MCC > 0.5 on the test set (Additional file 1: Table S3). The best performing model consisted of a total of three methylation markers, being one classifier: *ANKRD18CP*, and three predictors: *LHX8*, *EPB41L3* and *ANKRD18CP* (Additional file 1: Fig. S3). This model had an AUC of 0.83 in the training set and 0.84 in the test set (Fig. 4). The robustness score of the classifier was 591/1000. The robustness scores for the predictors were: 978/1000 for *LHX8*, 912/1000 for *EPB41L3* and 564/1000 for *ANKRD18CP,* with a mean of standard deviations of the coefficients per node of 0.15 and 0.24 (Additional file 1: Table S3). This shows that the model is very robust to detect CIN3+. The cut-off of the predicted probability to consider a sample CIN3+ was obtained based on the ROC curve of the train set, with a Youden index of 0.28. The sensitivity for this model to detect CIN3+ was 82% in the training set and 84% in the test set. The specificity was 74% in the training set and 71% in the test set. The predicted probabilities of CIN3+ per histological outcome in the full data are shown (Fig. 5). In the full data using this model, 19/45 (42%) CIN2 cases were predicted as CIN3+, 74/91 (81%) CIN3 cases were predicted as CIN3+ and all five (100%) cancer cases were predicted.

## Discussion

In this study, we found the most discriminative panel for CIN3+ detection, consisting of three markers: *ANKRD18CP, LHX8* and *EPB41L3*. This panel, identified by model-based recursive partitioning decision tree analysis and a robustness analysis showed an AUC of 0.83 in the training set and 0.84 in the test set, was robust and had a high sensitivity (84%) and specificity (71%) for the detection of CIN3+ in the test set. In addition, all cancer cases were detected as CIN3+ by this panel. This panel was created by analysing 15 discriminative host DNA methylation markers to detect CIN3+ based on a systematic literature search (sensitivity ≥ 70% and specificity ≥ 60%) on the same real-life cohort of self-samples obtained through the PBS in the North of the Netherlands.

We found that the methylation levels of all 15 tested markers in this study showed a significant increase with the severity of the underlying lesion, and for all tested markers the AUC’s of the ROC curves were significant for CIN3 + (*p* < 0.05). Nine markers had an AUC of ≥ 0.7. Consequently, six markers did not have an AUC of ≥ 0.7. This might be explained by the fact that some markers are selected based on their performance in a panel. In addition, some of the markers are only tested on cervical scrapes collected by the GP and not on self-samples. Previous research has already demonstrated that the performance of methylation markers on cervical scrapes and self-samples can be different [35]. This might be due to the fact that the two sample types differ in cellular composition. As in case of self-sampling, in addition to cervical indicator cells, mainly vaginal cells will be present. This can result in relative lower methylation levels in self-samples, in line with previous results [36]. However, our objective was to create a panel of methylation markers, which enables the detection of as many CIN3+ cases as possible. Therefore, the fact that some of the markers had a lower AUC as an individual marker might not be disadvantageous, as this methylation marker can still have added value in a panel.

The best panel of methylation markers for the detection of CIN3+ on self-samples found in our study consists of the three markers *LHX8, EPB41L3* and *ANKRD18CP*. *LHX8* has shown in previous studies to have an AUC of 0.83–0.89 in GP-collected material [28, 37]. In our study, *LHX8* has an AUC of 0.78 as an individual marker, and the lower AUC might be explained by the fact that this study is performed on self-samples. *ANKRD18CP* showed a sensitivity of 47–74% and specificity of 71% for CIN3+ in GP-collected material [23, 24]. This is in line with this study, as we also detected a lower AUC for *ANKRD18CP* as an individual marker, compared with the other markers present in our panel. The individual performance of *EPB41L3* has previously been described in self-samples. *EPB41L3* showed a 79% sensitivity with an 88% specificity in Evalyn® brush samples [36]. In the current study, *EPB41L3* has an AUC of 0.72 as an individual marker. However, the previous study was performed on a small cohort.

Some different panels of methylation markers have already been described in the literature. The combination of *ASCL1* and *LHX8* has shown a sensitivity of 77–83% and a specificity of 75–82% for CIN3+ in cervical scrapings [28, 37]. However, the second study, with the highest sensitivity and specificity (83%, 82%), did not include CIN2 cases, resulting in a higher specificity compared to the specificity of our best panel. There is one study describing the markers *LHX8* and *ASCL1* in hrHPV-positive self-samples. Both markers were combined with *ST6GALNAC5* and showed a sensitivity of 88% and a specificity of 81% in brush samples [38]. However, this study used a selected population (non-attending women), while the present study relies on real-life samples from the PBS. In addition, we did not combine *LHX8* and *ASCL1*, as *LHX8* and *ASCL1* are present in the same hierarchical cluster. For our decision tree model, we did not use combinations of markers from the same cluster for two reasons. First, when two markers are present in the same cluster, they will not add much extra information, as they are expected to show similar methylation profiles. Second, the decision tree model consists of logistic regression models at each terminal node. To avoid multicollinearity of the input predictors, it is important that markers are not correlated to each other, and thus not present in the same cluster. If the input predictors are correlated, this might have a negative impact on the regression, as this will result in a larger standard error of a logistic regression coefficient, and it will be less likely that this coefficient will be statistically significant [39].

In this study, we performed our modeling analysis to find the most optimal panel of methylation markers for the detection of CIN3+. In the Netherlands, in addition to CIN3+ lesions, treatment with surgical excision of CIN2 lesions is also an option. For this reason, we also evaluated the performance of the selected methylation markers for CIN2+. However, the CIN2 category is very heterogeneous, not only in its clinical behaviour, but also in the (epi)genetic profile. In a former study, we found that two-third of the CIN3 cases and only half of the CIN2 cases showed a cancer-like methylation-high pattern putatively related with the percentage of CIN lesions that will progress when left untreated [40]. We found that when we calculate the predicted probabilities using our decision tree model on the full dataset, 42% of the CIN2 had a probability score for CIN3+ and 81% of the CIN3 lesions had a probability score for CIN3+ showing again the likelihood of those lesions that might progress to cancer.

Currently in the Netherlands cytology is used as a triage test, the test has some limitations, as it is subjective, it requires a high level of skills and its inability of high-throughput testing [41]. There are some studies describing the performance of cytology as a triage test with sensitivity ranging between 63–92% and specificity between 49–72% for CIN3+ detection [12, 42–46]. However, most of these studies are randomized controlled trials and do not display the real screening population. A recent study, including women (more than 40,000) within the first 14 months of the new Dutch PBS for cervical cancer shows that cytology as a triage test has a sensitivity of 82% and a 76% specificity for CIN3+ detection [47].

In the Netherlands, women using the self-sampling device is increasing with a participation of 7.2% in 2017 to 22.1% in 2021 [17, 19]. Showing this might be a promising tool to improve participation.

A molecular triage test, such as methylation analysis can be directly performed on the same self-samples used for hrHPV testing, which makes methylation analysis appealing as a triage test. Especially because in the Netherlands 10% of the women do not go to the GP within 27 months after they receive an hrHPV-positive self-sampling result [17]. This results in an enormous delay before correct referral, but above all also loss in compliance to the PBS for cervical cancer.

In this study, we focused on obtaining at least a comparable sensitivity and specificity for CIN3+ on self-samples as cytology on GP samples. Therefore, we set stricter criteria for the sensitivity as for the specificity. In addition, in this study we used a selected population, with more cases with an abnormal cytology result. This means that the specificity for cytology is also lower in our population and that we could set lower criteria for the specificity of the methylation test in this population. However, we still found that the performance of our best panel in our discovery study is very similar to the performance of cytology [12, 42–47]. Taken together, our data suggest that our methylation test might be a potential option to replace cytology. Clinical validation on an independent series of consecutive samples is needed to confirm the performance of our methylation panel.

A major strength of this study is that all 15 methylation markers identified by different research groups are analysed on the same cohort of self-samples on the same platform, obtained in an unique setting, namely through the PBS programme in the North of the Netherlands. This is exceptional as, most of the studies evaluating DNA methylation markers have been performed in referral or case–control settings, meaning that a selected population of women is participating, often consisting of non-attending women. In addition, in most studies only a few makers are analysed simultaneously on the same population, while in this study we analyse all potential methylation markers on the same population.

Moreover, another strength is that our data analysis uses model-based recursive partitioning, followed by a robustness analysis. Basic logistic regression models, which are generally used [28, 40], provide a single coefficient for each predictor assuming that the predictor is associated to the response variable in the same way irrespective of selection of patients. When the association between the predictor and the response variable varies significantly with respect to the selected subset of patients, then the coefficient obtained from logistic regression does not completely reflect the real relation between the variables. However, a model-based recursive partitioning decision tree analysis can handle this issue by splitting the group of patients using classifiers and thereafter conducting logistic regression at each terminal node. Another way to get rid of this problem is to apply classification and regression tree (CART) to obtain a model for disease status, as for example used by Verlaat et al. [38]. CART classifies the patients into multiple groups without conducting a regression at the terminal nodes. The use of classification without regression leads to less robust sensitivity and specificity of the model due to lesser choice of possible predicted probabilities. In addition, execution of a split in the model-based tree indicates a parameter instability in the original model, showing that a single logistic regression model on full data would be too simple to explain the data [34, 48, 49].

One limitation of our study is the overrepresentation of CIN2 cases in the < CIN3 group of the study population, which might lead to lower specificity than found in a representative cohort. However, the distribution of CIN3 and cancer in the CIN3+ group is representative of the screened Dutch population, resulting in an adequate sensitivity.

The relative small sample size requires validation of the selected panel on a larger representative cohort of consecutive hrHPV-positive self-samples collected form the Dutch PBS with sufficient power to confirm the performance of our best panel.

Furthermore, our analysis is focused on host DNA methylation markers that could be tested on the same platform, under the same conditions. This resulted in the fact that we did not analyse markers that are part of commercial assays, for example the QIAsure and Gyntect® assay, as these assays could not be performed on our platform. So, we cannot exclude that these markers have additional value to the ones we found.

## Conclusion

In this study for the first time, 15 non-commercial host DNA methylation markers with published sensitivity ≥ 70% and specificity ≥ 60% for CIN3 + were analysed on the same cohort of hrHPV-positive self-samples collected through the PBS. We found a three-marker panel of *ANKRD18CP, LHX8* and *EPB41L3*, which showed a sensitivity of 82% and a specificity of 74% in the training set, and a sensitivity of 84% and a specificity of 71% in the test set. This promising three marker panel might, after clinical validation in an independent series, be implemented in the PBS in the Netherlands. This panel can be tested on the same self-samples used for hrHPV testing and would not require any further GP visit and will result in faster correct referral to the gynaecologist.

## Supplementary Information

**Additional file 1: Table S1** Selected host DNA methylation markers. **Figure S1** Methylation levels of the 15 selected markers. **Figure S2** ROC curve of the 15 individual methylation markers. **Table S2** AUC of the ROC analyses of the 15 individual methylation markers for the detection of CIN3+ and CIN2+. **Figure S3** Decision tree model of the best panel of methylation markers to detect CIN3+. **Table S3** List with models that fulfilled the set criteria (sensitivity > 80% and specificity > 65% and Matthew's correlation coefficient (MCC) > 0.5 on the test set) and the robustness score for the classifiers and the predictors.

## Data Availability

All data are available on request.
