# Supplementary material for: Identification of a methylation panel as an alternative triage to detect CIN3+ in hrHPV-positive self-samples from the population-based cervical cancer screening programme
Source: Clin Epigenetics. 2023 Jun 15;15:103. doi: 10.1186/s13148-023-01517-6 (PMC10273737; doi:10.1186/s13148-023-01517-6)
Supplement: Supplementary file 1 — Additional file 1: Table S1 Selected host DNA methylation markers. Figure S1 Methylation levels of the 15 selected markers. Figure S2 ROC curve of the 15 individual methylation markers. Table S2 AUC of the ROC analyses of the 15 individual methylation markers for the detection of CIN3+ and CIN2+. Figure S3 Decision tree model of the best panel of methylation markers to detect CIN3+. Table S3 List with models that fulfilled the set criteria (sensitivity > 80% and specificity > 65% and Matthew's correlation coefficient (MCC) > 0.5 on the test set) and the robustness score for the classifiers and the predictors. [file 13148_2023_1517_MOESM1_ESM.docx]

**Supplementary Information

Supplementary methods
Model based recursive partitioning (MOB) as a predictive model***1. Decision tree analysis.*

Model based recursive partitioning (MOB) was conducted to obtain a decision tree for predicting CIN3+ status (binary levels, 0 or 1) of a self-sample using ∆Ct values of the methylation markers as predictors and/or classifiers. All the 15 methylation markers were used as classifiers and different combinations of a subset of markers were used as predictors in different predictive models (explained in detail below). The MOB was built with a recursive process. At each recursive step, logistic regression analyses were conducted in two subgroups obtained using a single ∆Ct value threshold for a classifier marker. All ∆Ct value thresholds for all methylation markers were evaluated on the degree of parameter instability of the predictor markers in two subgroups (assessed with a fluctuation test). Next, the significant classifier marker and its ∆Ct value threshold for the corresponding node were selected based on the most significant fluctuation test. This recursive step was repeated separately for each of the two sample subgroups resulting from splitting the samples based on this significant classifier marker and its ∆Ct value threshold. A recursive step stopped if the Bonferroni-corrected significance level (α) was above 0.05 for all thresholds of all input classifiers or if the number of total samples in the terminal nodes dropped below 25 [1–3].

To estimate the performance of the predictive model, the self-samples and corresponding ∆Ct values for all the methylation markers were divided into a train set (80%) and test set (20%) using stratified random sampling (without replacement). The train and test set were obtained using stratification on histological diagnosis so that the percentage for all the different histological diagnoses (e.g. CIN0, 1, 2 etc.) was similar in the train and test set. Decision tree analysis was conducted using the mob function from the party package version 1.3-9 in R.

*2. Framework of using methylation markers as predictors in MOB to find the best predictive model.*
To find the best combination of markers predicting CIN3+ status of women, a framework was used to assess the performance of each MOB. The framework consists of the following steps:

1. Methylation markers were grouped based on the clustering.
   1. Hierarchical clustering analysis was conducted on ∆Ct values of methylation markers using 1 – spearman correlation as distance and ward.D2 method. The dendrogram was cut at a height of 0.5 to obtain clusters.
   2. Only one marker per cluster was eligible to be used as an input predictor in the MOB. Different combinations of input predictor markers were obtained so that the markers in a particular combination were belonging to different clusters. The maximum number of input predictors in a particular combination was set to six, as our aim is to create a quantitative multiplex PCR, and more than six markers will not be workable.
   3. A matrix with all possible combinations of input predictors was created.
2. For each possible combination of input predictors:
   1. A decision tree with fitted logistic regression at every node was created.
   2. The predicted probability of being CIN3+ for each sample was obtained.
   3. The Area under curve of ROC ‘AUC’, ‘sensitivity’, ‘specificity’, ‘Matthew's correlation coefficient (MCC)’ were obtained for as well the train and test set. For the ‘sensitivity’, ‘specificity’, ‘Matthew's correlation coefficient’, ‘precision recall’ and ‘F1’, the cut-off based on the maximum value of the Youden index (sensitivity + specificity -1) corresponding to the ROC curve on the train set was used.
   4. The list of predictors which were significantly associated to CIN3+ status and the list of significant classifiers were obtained.
   5. The logistic regression coefficients of corresponding input predictors in all the terminal nodes were obtained.
3. The models that fulfilled the following criteria were selected for further investigation:
   a. sensitivity >80% and specificity >65% and MCC >0.5 on the test set.

*3. Robustness analysis of selected decision trees*.
Robustness analysis was performed for these decision trees individually who passed the selection criteria (see above). MOB was conducted separately 1,000 times using random 80% of the samples as training set and 20% as test set for each of the selected trees.

To quantify the robustness of each of the selected decision trees, the following steps were conducted:

1. For each of the 1,000 iterations:

1. The input predictors for all the iterations were set as the exact list of input predictors in the corresponding selected decision tree. All the 15 markers were used as classifiers.
2. The number of times the same significant classifier (as in the selected decision tree) was significantly classifying the training set out of 1,000 times was calculated to evaluate the robustness of the classifier in the selected decision tree model.
3. The mean of standard deviations (sd’s) of regression coefficients for all the predictors per node were calculated and the mean of sd per model (for both nodes) was calculated to judge the robustness of the coefficients of the predictors in each individual logistic regression model at terminal nodes of the decision tree.
4. The number of times the coefficients for the individual predictors were significant in each individual logistic regression model at terminal nodes of the decision tree was calculated (robustness score predictors).

2. Robust models were selected using the following criteria:

a. A robustness score (indicating markers significantly appearing as a classifier in the tree) of above 500 out of 1,000 for the classifier.

b. All the individual predictors needed to have a robustness score (indicating markers significantly appearing as a predictor in the tree) of above 500 out of 1,000.
c. The mean of the standard deviations of the coefficients needed to be below 1 (indicating all the coefficients of predictors deviated least due to different subsets of input dataset).

**Supplementary Table 1.** Selected host DNA methylation markers.
*Criteria: in at least one study a sensitivity of ≥70% and a specificity of ≥60%, as a single marker or the marker is present in a panel which fulfils these criteria.*

| Gene | Reference | Sequences received from |
| --- | --- | --- |
| ANKRD18CP | Boers et al., 2016;  van Leeuwen et al., 2019 | UMCG |
| ASCL1 | Verlaat et al., 2018;  Dick et al., 2020;  Verhoef et al., 2022 | Self-screen B.V. |
| C13ORF18 | Boers et al., 2016;  van Leeuwen et al., 2019 | UMCG |
| EPB41L3 | Boers et al., 2014;  Boers et al., 2016;  Eijsink et al., 2011;  van Leeuwen et al., 2019 | UMCG |
| GHSR | Verlaat et al., 2017; Dick et al., 2020; | Steenbergen, Vu |
| JAM3 | Boers et al., 2014;  Boers et al 2016;  Eijsink et al., 2011;  Yin et al., 2015 | UMCG |
| LHX8 | Verlaat et al., 2018;  Dick et al., 2020;  Verhoef et al., 2022 | Self-screen B.V. |
| PAX1 | Chang et al., 2015;  Kan et al., 2014;  Chang et al., 2021;  Zhang et al., 2020 | Lai, Taipei |
| POU4F3 | Kocsis et al., 2017;  Pun et al., 2015 | Lai, Taipei |
| SOX1 | Chang et al., 2015;  van Leeuwen et al., 2019; Wang et al., 2016;  Zhang et al., 2020 | UMCG |
| SST | Verlaat et al., 2017; Dick et al., 2020; | Steenbergen, Vu |
| ST6GALNAC5 | Verlaat et al., 2018; Dick et al., 2020; | Steenbergen, Vu |
| ZIC1 | Verlaat et al., 2017;  Dick et al., 2020 | Steenbergen, Vu |
| ZNF582 | Chang et al., 2015;  Lin et al., 2014 | Lai, Taipei |
| ZSCAN1 | van Leeuwen et al., 2019 | UMCG |


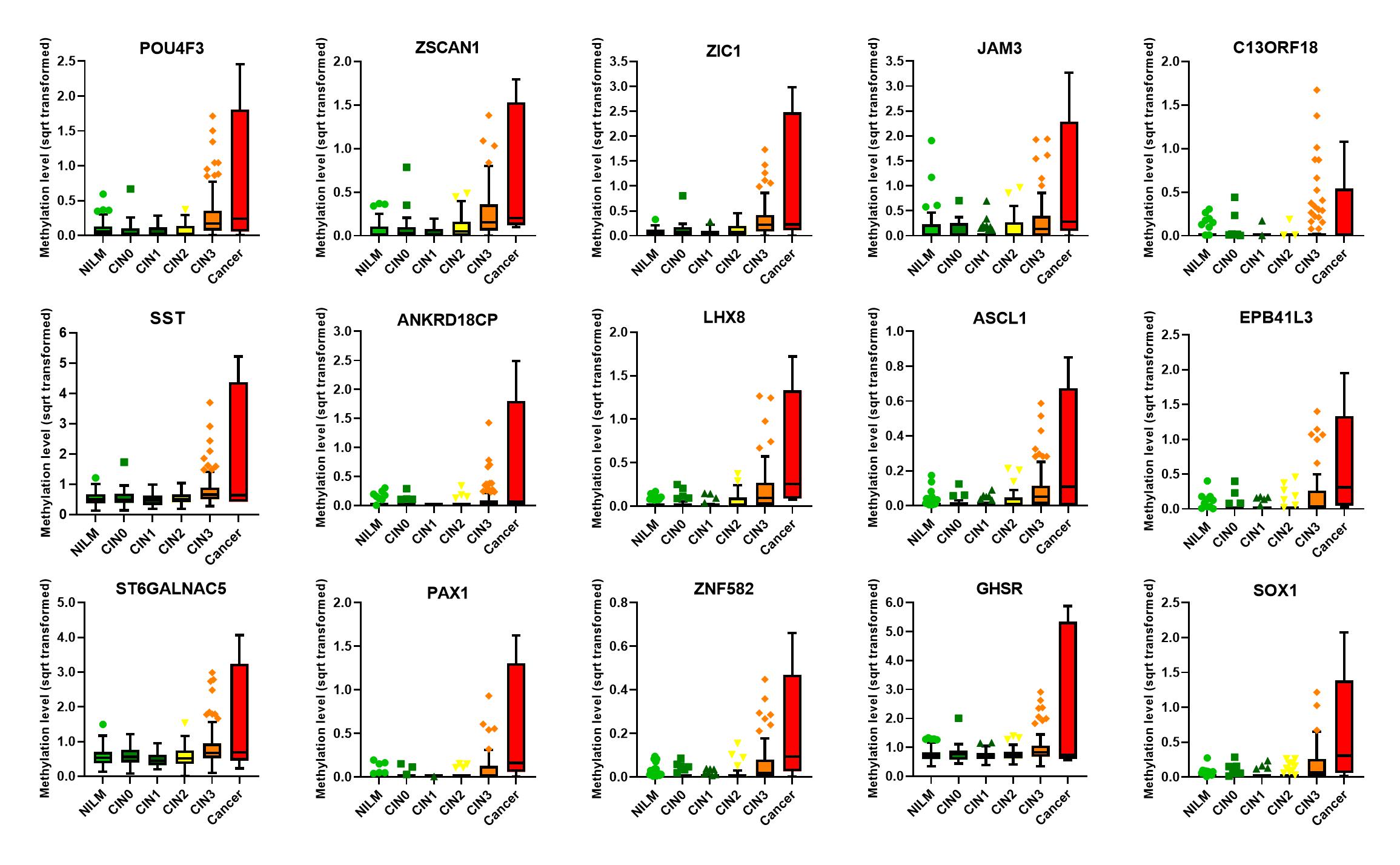


**Supplementary Figure 1.** Methylation levels of the 15 selected markers.
*Methylation levels across histological subgroups (NILM N=94, CIN0 N=31, CIN1 N=38, CIN2 N=45, CIN3 N=91, cancer N=5) for all the different methylation markers.* *All markers were discriminative between CIN2+ and <CIN2 (p<0.05) and between CIN3+ and <CIN3 (p<0.001).*


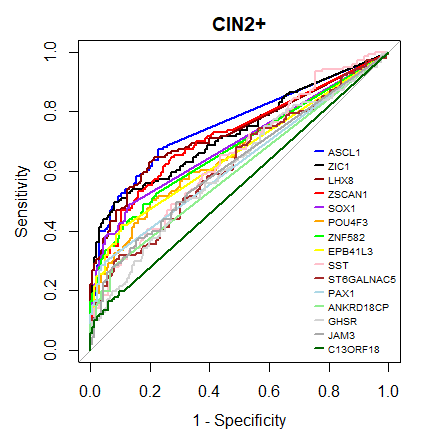


**Supplementary Figure 2.** ROC curve of the 15 individual methylation markers.
*ROC curves for ∆Ct values of the 15 individual methylation markers for the detection of CIN2+.*

**Supplementary Table 2**. AUC of the ROC analyses of the 15 individual methylation markers for the detection of CIN3+ and CIN2+.

| **Methylation markers** | **Area under the curve CIN3+** | **Asymptotic sign. *** | **Asymptotic 95% Confidence Interval** | | **Area under the curve CIN2+** | **Asymptotic sign. *** | **Asymptotic 95% Confidence Interval** | |
| --- | --- | --- | --- | --- | --- | --- | --- | --- |
| **ASCL1** | .806 | .000 | .749 | .863 | .758 | .000 | .702 | .814 |
| **ZIC1** | .787 | .000 | .729 | .844 | .728 | .000 | .670 | .786 |
| **LHX8** | .781 | .000 | .721 | .841 | .733 | .000 | .675 | .792 |
| **ZSCAN1** | .762 | .000 | .701 | .823 | .719 | .000 | .660 | .778 |
| **SOX1** | .746 | .000 | .680 | .812 | .693 | .000 | .632 | .754 |
| **POU4F3** | .745 | .000 | .682 | .808 | .663 | .000 | .601 | .725 |
| **ZNF582** | .731 | .000 | .665 | .798 | .679 | .000 | .618 | .740 |
| **EPB41L3** | .722 | .000 | .655 | .790 | .662 | .000 | .600 | .724 |
| **SST** | .700 | .000 | .637 | .764 | .652 | .000 | .591 | .713 |
| **ST6GALNAC5** | .676 | .000 | .609 | .743 | .627 | .000 | .564 | .690 |
| **PAX1** | .676 | .000 | .605 | .746 | .626 | .000 | .562 | .690 |
| **ANKRD18CP** | .646 | .000 | .575 | .718 | .604 | .002 | .539 | .668 |
| **GHSR** | .645 | .000 | .576 | .714 | .612 | .001 | .548 | .676 |
| **JAM3** | .635 | .000 | .565 | .705 | .625 | .000 | .561 | .688 |
| **C13ORF18** | .587 | .015 | .514 | .659 | .549 | .144 | .483 | .614 |

*** Null hypothesis: true area = 0.5**


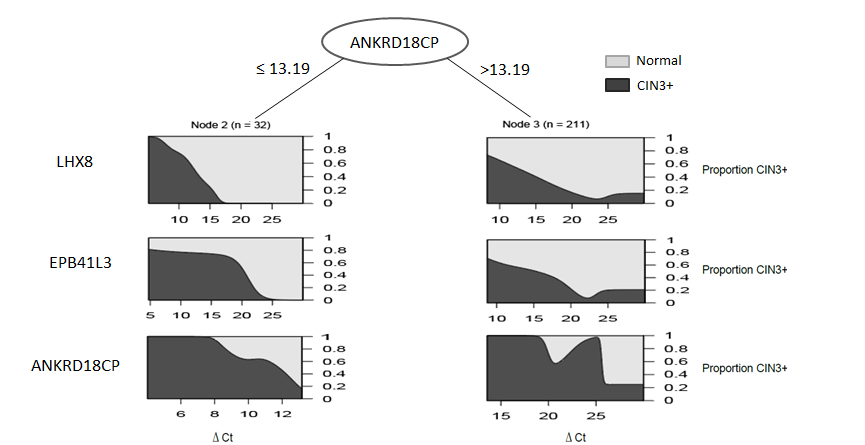


**Supplementary Figure 3.** Decision tree model of the best panel of methylation markers to detect CIN3+.
*This model shows whether a sample is regarded as CIN3+/<CIN3+, respectively. The* ∆Ct *value of the first methylation marker (the classifier, ANKRD18CP) was used to decide whether the sample belongs to the left or the right node. Both nodes consist of a different linear model. By adding the ∆Ct values of the markers (the predictors, LHX8, EPB41L3 and ANKRD18CP) to this linear model, the odds ratio can be calculated. The graphs show the proportion of CIN3+ lesions at a certain ∆Ct value for each methylation marker.*

**Supplementary Table 3.** List with models that fulfilled the set criteria (sensitivity >80% and specificity >65% and Matthew’s correlation coefficient (MCC) >0.5 on the test set) and the robustness score for the classifiers and the predictors. The models are ranked based on their robustness scores, for as well the classifiers and the predictors. The model which fulfilled the robustness criteria (score of ≥500) for as well the classifiers and the predictors and mean of standard deviations of the coefficients below 1 is put on top.

| Model | Predictors | Classifiers | AUC train | AUC test | Specificity train | Sensitivity train | Specificity test | Sensitivity test | MCC train | MCC test | Robustness classifier | Mean of sd of coefficients | Robustness predictors |
| --- | --- | --- | --- | --- | --- | --- | --- | --- | --- | --- | --- | --- | --- |
| 1 | LHX8, EPB41L3, ANKRD18CP | ANKRD18CP | 0.83 | 0.84 | 0.74 | 0.82 | 0.71 | 0.84 | 0.52 | 0.52 | 591 | 0.20 | LHX 978 EPB41L3 912 ANKRD18CP 564  Average of predictors: 818 |
| 2 | SST, JAM3, ASCL1, PAX1 | PAX1 | 0.83 | 0.85 | 0.72 | 0.79 | 0.79 | 0.84 | 0.48 | 0.59 | 987 | 83 | SST 56 JAM3 4 ASCL1 1000 PAX1 703 Average of predictors: 441 |
| 3 | SST, ST6GALNAC5, ASCL1, C13ORF18 | C13ORF18 | 0.82 | 0.87 | 0.68 | 0.83 | 0.74 | 0.89 | 0.48 | 0.59 | 952 | 36 | SST 549 ST6GALNAC5 9 ASCL1 997 C13ORF18 0  Average of predictors: 389 |
| 4 | SST, JAM3, ASCL1, C13ORF18, PAX1 | C13ORF18 | 0.82 | 0.90 | 0.75 | 0.78 | 0.81 | 0.84 | 0.50 | 0.62 | 340 | 67 | SST 185 JAM3 19 ASCL1 997 C13ORF18 14 PAX1 537 Average of predictors: 350 |
| 5 | SST, ST6GALNAC5, JAM3, ASCL1, C13ORF18, PAX1 | C13ORF18 | 0.82 | 0.90 | 0.75 | 0.78 | 0.81 | 0.89 | 0.59 | 0.66 | 229 | 11 | SST 140 ST6GALNAC5 0 JAM3 14 ASCL1 996 C13ORF18 12 PAX1 504 Average of predictors: 278 |
| 6 | GHSR, JAM3, C13ORF18, ANKRD18CP | ZIC1, ASCL1, GHSR | 0.88 | 0.84 | 0.72 | 0.88 | 0.79 | 0.84 | 0.56 | 0.59 | 112 | 5 | GHSR 115 JAM3 69 C13ORF18 86 ANKRD18CP 265 Average of predictors: 134 |
| 7 | GHSR, JAM3, C13ORF18 | ZIC1, ASCL1, GHSR | 0.88 | 0.84 | 0.72 | 0.87 | 0.79 | 0.84 | 0.55 | 0.59 | 99 | 15 | GHSR 113 JAM3 60 C13ORF18 86 Average of predictors: 86 |

**References**

1. Zeileis A, Hothorn T, Hornik K. Model-based recursive partitioning. J Comput Graph Stat. 2008;17:492–514.

2. Seibold H, Zeileis A, Hothorn T. Model-Based Recursive Partitioning for Subgroup Analyses. Int J Biostat. 2016;12:45–63.

3. Hothorn T, Hornik K, Zeileis A. Unbiased recursive partitioning: A conditional inference framework. J Compu Graph Stat. 2006;15:651–74.

4. Boers A, Wang R, van Leeuwen RW, Klip HG, de Bock GH, Hollema H, et al. Discovery of new methylation markers to improve screening for cervical intraepithelial neoplasia grade 2/3. Clin Epigenetics. Clin Epigenetics; 2016;8:1–16.

5. van Leeuwen RW, Oštrbenk A, Poljak M, van der Zee AGJ, Schuuring E, Wisman GBA. DNA methylation markers as a triage test for identification of cervical lesions in a high risk human papillomavirus positive screening cohort. Int J Cancer. 2019;144:746–54.

6. Verlaat W, Snoek BC, Heideman DAM, Wilting SM, Snijders PJF, Novianti PW, et al. Identification and validation of a 3-gene methylation classifier for hpv-based cervical screening on self-samples. Clin Cancer Res. 2018;24:3456–64.

7. Dick S, Verhoef L, de Strooper LMA, Ciocănea-Teodorescu I, Wisman GBA, Meijer CJLM, et al. Evaluation of six methylation markers derived from genome-wide screens for detection of cervical precancer and cancer. Epigenomics. 2020;12:1569–78.

8. Verhoef L, Bleeker MCG, Polman N, Steenbergen RDM, Meijer CJLM, Melchers WJG, et al. Performance of DNA methylation analysis of ASCL1, LHX8, ST6GALNAC5, GHSR, ZIC1 and SST for the triage of HPV-positive women: Results from a Dutch primary HPV-based screening cohort. Int J Cancer. 2022;150:440–9.

9. Yang N, Eijsink JJH, Lendvai Á, Volders HH, Klip H, Buikema HJ, et al. Methylation markers for CCNA1 and C13ORF18 are strongly associated with high-grade cervical intraepithelial neoplasia and cervical cancer in cervical scrapings. Cancer Epidemiol Biomarkers Prev. 2009;18:3000–7.

10. Eijsink JJH, Yang N, Lendvai A, Klip HG, Volders HH, Buikema HJ, et al. Detection of cervical neoplasia by DNA methylation analysis in cervico-vaginal lavages, a feasibility study. Gynecol Oncol. 2011;120:280–3.

11. Boers A, Bosgraaf RP, van Leeuwen RW, Schuuring E, Heideman DA, Massuger LF, et al. DNA methylation analysis in self-sampled brush material as a triage test in hrHPV-positive women. Br J Cancer. 2014;111:1095–101.

12. Verlaat W, Snijders PJF, Novianti PW, Wilting SM, De Strooper LMA, Trooskens G, et al. Genome-wide DNA methylation profiling reveals methylation markers associated with 3q gain for detection of cervical precancer and cancer. Clin Cancer Res. 2017;23:3813–22.

13. Yin A, Zhang Q, Kong X, Jia L, Yang Z, Meng L, et al. JAM3 methylation status as a biomarker for diagnosis of preneoplastic and neoplastic lesions of the cervix. Oncotarget. 2015;6:44373–87.

14. Kan YY, Liou YL, Wang HJ, Chen CY, Sung LC, Chang CF, et al. PAX1 methylation as a potential biomarker for cervical cancer screening. Int J Gynecol Cancer. 2014;24:928–34.

15. Lin H, Chen TC, Chang TC, Cheng YM, Chen CH, Chu TY, et al. Methylated ZNF582 gene as a marker for triage of women with Pap smear reporting low-grade squamous intraepithelial lesions - A Taiwanese Gynecologic Oncology Group (TGOG) study. Gynecol Oncol. 2014;135:64–8.

16. Pun PB, Liao YP, Su PH, Wang HC, Chen YC, Hsu YW, et al. Triage of high-risk human papillomavirus-positive women by methylated POU4F3. Clin Epigenetics. 2015;7:1–8.

17. Chang CC, Huang RL, Liao YP, Su PH, Hsu YW, Wang HC, et al. Concordance analysis of methylation biomarkers detection in self-collected and physician-collected samples in cervical neoplasm. BMC Cancer. 2015;15.

18. Chang CL, Ho SC, Su YF, Juan YC, Huang CY, Chao AS, et al. DNA methylation marker for the triage of hrHPV positive women in cervical cancer screening: Real-world evidence in Taiwan. Gynecol Oncol. 2021;161:429–35.

19. Zhang L, Yu J, Huang W, Zhang H, Xu J, Cai H. A sensitive and simplified classifier of cervical lesions based on a methylation-specific PCR assay: A Chinese cohort study. Cancer Manag Res. 2020;12:2567–76.

20. Kocsis A, Takács T, Jeney C, Schaff Z, Koiss R, Járay B, et al. Performance of a new HPV and biomarker assay in the management of hrHPV positive women: Subanalysis of the ongoing multicenter TRACE clinical trial (n > 6,000) to evaluate POU4F3 methylation as a potential biomarker of cervical precancer and cancer. Int J Cancer. 2017;140:1119–33.

21. Wang R, van Leeuwen RW, Boers A, Klip HG, Meyer T de, Steenbergen RDM, et al. Genome-wide methylome analysis using MethylCap-seq uncovers 4 hypermethylated markers with high sensitivity for both adeno- and squamous-cell cervical carcinoma. Oncotarget. 2016;7:80735–50.
